# Protective effect of the poly(ADP-ribose) polymerase inhibitor PJ34 on mitochondrial depolarization-mediated cell death in hepatocellular carcinoma cells involves attenuation of c-Jun N-terminal kinase-2 and protein kinase B/Akt activation

**DOI:** 10.1186/1476-4598-11-34

**Published:** 2012-05-14

**Authors:** Balazs Radnai, Csenge Antus, Boglarka Racz, Peter Engelmann, Janos Krisztian Priber, Zsuzsanna Tucsek, Balazs Veres, Zsuzsanna Turi, Tamas Lorand, Balazs Sumegi, Ferenc Gallyas

**Affiliations:** 1 Department of Biochemistry and Medical Chemistry, University of Pécs Medical School, 12 Szigeti st., H-7624, Pécs, Hungary; 2 Department of Immunology & Biotechnology, University of Pécs Medical School, 12 Szigeti st., H-7624, Pécs, Hungary; 3 Nuclear-Mitochondrial Interactions Research Group, Hungarian Academy of Sciences, PO Box 1000, H-1245, Budapest, Hungary; 4 Szentagothai Research Center, 34 Ifjusag st., H-7624, Pécs, Hungary

**Keywords:** Poly(ADP-ribose)-polymerase (PARP), Mitogen activated protein kinase (MAPK), c-Jun N-terminal kinase (JNK), Protein kinase B (PKB/Akt), Mitochondrial depolarization

## Abstract

**Background:**

2,4-Dimethoxyphenyl-*E*-4-arylidene-3-isochromanone (IK11) was previously described to induce apoptotic death of A431 tumor cells. In this report, we investigated the molecular action of IK11 in the HepG2 human hepatocellular carcinoma cell line to increase our knowledge of the role of poly (ADP-ribose)-polymerase (PARP), protein kinase B/Akt and mitogen activated protein kinase (MAPK) activation in the survival and death of tumor cells and to highlight the possible role of PARP-inhibitors in co-treatments with different cytotoxic agents in cancer therapy.

**Results:**

We found that sublethal concentrations of IK11 prevented proliferation, migration and entry of the cells into their G2 phase. At higher concentrations, IK11 induced reactive oxygen species (ROS) production, mitochondrial membrane depolarization, activation of c-Jun N-terminal kinase 2 (JNK2), and substantial loss of HepG2 cells. ROS production appeared marginal in mediating the cytotoxicity of IK11 since N-acetyl cysteine was unable to prevent it. However, the PARP inhibitor PJ34, although not a ROS scavenger, strongly inhibited both IK11-induced ROS production and cell death. JNK2 activation seemed to be a major mediator of the effect of IK11 since inhibition of JNK resulted in a substantial cytoprotection while inhibitors of the other kinases failed to do so. Inhibition of Akt slightly diminished the effect of IK11, while the JNK and Akt inhibitor and ROS scavenger trans-resveratrol completely protected against it.

**Conclusions:**

These results indicate significant involvement of PARP, a marginal role of ROS and a pro-apoptotic role of Akt in this system, and raise attention to a novel mechanism that should be considered when cancer therapy is augmented with PARP-inhibition, namely the cytoprotection by inhibition of JNK2.

## Background

PARP-1 is a nuclear enzyme present in eukaryotes in a high copy number. One of its major physiological functions is responding to single- and double-strand DNA breaks and facilitating DNA repair. Inhibition of PARP-1 sensitizes cells to DNA-damaging agents [1] indicating its potentiality in facilitating tumor therapy. Furthermore, it was shown that cells deficient in breast cancer associated gene-1 and −2 (BRCA1/2) are extremely sensitive to PARP-1 inhibition because of the defective double-strand DNA break repair [2] in these cells. Therefore, PARP inhibition is considered as a useful therapeutic strategy not only for the treatment of BRCA mutation-associated tumors, but also for the treatment of a wider range of tumors bearing a variety of deficiencies in the homologous recombination DNA repair pathway [3]. PARP inhibitors were also found to protect cells and tissues in different pathophysiological conditions [4] by various mechanisms including activation of the cytoprotective phosphatidylinositol-3 kinase (PI3K)-Akt pathway [5] that could even impair the efficacy of tumor therapy and mediate drug-resistance [6,7].

Besides the Akt pathway, PARP activation was associated with all three branches of mitogen activated protein kinases (MAPKs); the *c-jun* N-terminal kinase (JNK) [8], the p38 [8] and the extracellular signal regulated kinase (ERK) [9]. The latter is the main transducer of growth stimuli [9]; however, its role in the apoptosis inducing mechanism of cytotoxic agents appears to be more complex. On one hand, inhibition of ERK1/2 activity has been shown to increase the sensitivity of ovarian carcinoma cells against cisplatin [10], but on the other hand, activation of ERK1/2 was found to be required in cisplatin-induced apoptosis e.g. in renal proximal tubule cells [11]. The role of JNK and p38 cascades seems more straightforward. Mostly, they are associated with mediating the apoptotic signal, and their activation leads to cell death in various stress situations such as oxidative stress and inflammation [12].

Recently, we proposed that PARP activation in oxidative stress leads to suppression of MAPK phosphatase-1 (MKP-1) and thereby to the activation of p38 and JNK [13]. Activation of PARP and/or MAPKs could lead to mitochondrial depolarization [8,9,14]. Depolarization can result in the release of mitochondrial intermembrane proteins, triggering apoptosis, or in the permeability transition pore-dependent failure of ATP generation, leading to necrosis [15]. Accordingly, various mediators and regulators of mitochondrial depolarization-dependent cell death were suggested as targets in tumor therapy since mitochondrial mechanisms could facilitate either reversion of apoptotic resistance [16] or induction of necrosis via activation of permeability transition in the apoptosis-resistant tumor cells [17,18].

2,4-Dimethoxyphenyl-*E*-4-arylidene-3-isochromanone (IK11) was previously described to induce PARP cleavage dependent apoptosis in A431 tumor cell with high efficacy [19]. This finding suggested that the mechanism of IK11 induced cell death could be different from that of other substances used in previous studies. Although excessive over-activation of PARP is generally associated with necrotic cell death, over-activation of a lower extent could trigger apoptosis [4]. On the other hand, PARP cleavage is considered as an early indicator of the caspase dependent apoptotic process. Therefore, it seemed worth investigating how the PARP inhibitor PJ34 affected the IK11 induced cell death process. To this end, we determined the effect of IK11 on cell migration, apoptosis, necrosis, mitochondrial depolarization, reactive oxygen species (ROS) production as well as Akt and MAPK activation in HepG2 human hepatocellular carcinoma cells. Furthermore, we studied how inhibitors of PARP and intracellular kinase signaling pathways, and the antioxidant N-acetyl cysteine (NAC) affected the IK11 induced cell death process.

## Materials and methods

### Reagents

Primary antibodies, namely anti-phospho-Akt (S473), anti-phospho-p38 MAP-Kinase (T180/Y182), anti-phospho-p44/42 MAPK (T202/Y204) were from Cell Signaling Technology, anti-phospho-JNK (T183/Y185) from R&D System and anti-glyceraldehyde 3-phosphate dehydrogenase (GAPDH) we obtained from Millipore. The used kinase pathway inhibitors, namely Akt pathway inhibitors (LY294002, Akt inhibitor IV.) and JNK inhibitor (SP600125) were purchased from Calbiochem. All other substances including trans-resveratrol and PJ34 were from Sigma-Aldrich. IK11, was synthesized by us [20] and was dissolved in dimethyl-sulphoxide (DMSO) at 1000 times of the final concentration used. HO3089 [21] and L2286 [22] were kind gift of professor Kalman Hideg Department of Organic and Pharmacological Chemistry, University of Pecs Medical School, Pecs, Hungary.

### Cell culture

HepG2 human hepatocellular carcinoma cells obtained from European Collection of Cell Cultures were cultured in 5% CO_2_ at 37°C in Dulbecco’s Modified Eagle’s Medium supplemented with 10% fetal calf serum. Cells were seeded at a starting density of 2 × 10^4^ cells/well in a 96-well plate for viability and ROS production assays, or of 2 × 10^6^ cells/well in a 6-well plate for immunoblotting and determination of cell morphology. Full confluent 6 well plates were used for migration assay.

### Silencing of PARP by siRNA technique

HepG2 cells were transiently transfected with siRNA designed for PARP suppression by the manufacturer (Santa Cruz Biotechnology, Santa Cruz, CA) in Opti-MEM I Reduced Serum Medium (Invitrogen) using Lipofectamine 2000. For an effective suppression of PARP, the transfection step was repeated twice with a 48-h interval between the transfections, and the experiments on the cells were performed 40 h after the third transfection.

### Determination of intracellular reactive oxygen species

Intracellular ROS were determined using the oxidation-sensitive 2,4 dichlorodihydrofluorescein-diacetat (C-400) fluorescent dye. Cells were seeded into 96-well plates and cultured overnight. After subjecting the cells to the treatment indicated in the figure legends, medium was replaced to a fresh one containing 2 μg/ml C400. Incubation was continued for an additional 2 hrs to allow oxidation of C-400 by the endogenous ROS. Fluorescence of oxidized C-400 was excited at 485 nm and the evoked emission was measured at 555 nm by using a FLUOstar Optima fluorescent plate-reader. All experiments were run in at least 6 parallels and repeated three times.

### Cell viability assay

Cells were seeded and treated as for ROS determination. After the treatment, medium was replaced to a fresh one containing 0.5% MTT. Incubation was continued for an additional 3 h, and the reduction of MTT to formasan was terminated by adding isopropanol containing 0.4% HCl. The concentration of the water-insoluble formasan dye was proportional to the number of living cells. After dissolving the dye in the acidified isopropanol, the absorption was measured with an Anthos Labtech 2010 plate-reader at 550 nm wavelength. All experiments were run in 6 parallels and repeated three times.

### Immunoblot analysis

The cells were seeded into a 6-well plate and cultured overnight. After subjecting the cells to the treatment indicated in the figure legends for 6 h, the cells were harvested in ice-cold lysis buffer containing 0.5 mM sodium metavanadate, 1 mM ethylenediaminetetraacetic acid (EDTA), and protease inhibitor mixture in phosphate-buffered saline (PBS). The proteins were precipitated by trichloroacetic acid, washed three times with −20°C acetone, and subjected to sodium-dodecylsulphate polyacrylamide gel electrophoresis. Proteins (20 μg/lane) were separated on 12% gels and then transferred to nitrocellulose membranes. The membranes were blocked in 5% low fat milk for 1 h at room temperature and were exposed to the primary antibodies at 4°C overnight at a dilution of 1:1000 in blocking solution. Appropriate horseradish peroxidase-conjugated secondary antibodies were used for 2 h at room temperature in 1:5000 dilution. Peroxidase labeling was visualized with enhanced chemiluminescence using the SuperSignal West Pico chemiluminescent substrate (Pierce Chemical).

### Monolayer wound healing assay

HepG2 cells were seeded in six well plates and cultured until reaching full confluency. Then two perpendicular lines were drawn at the bottom of the well with a 1000 μl pipette tip. These lines served as marks for the wound areas to be analyzed. Then the medium was changed to a fresh one containing 1 μM IK11 for an additional 24 hrs incubation before taking images from a section of the wounds directly above the intercept of the two lines by a Zeiss Axiovert 25 microscope equipped with a ProgRes C12 Plus CCD camera using a 10x phase-contrast objective. All experiments were repeated three times.

### JC-1 assay for fluorescent microscopy

Mitochondrial membrane potential (∆Ψm) was measured using the mitochondrial membrane potential specific fluorescent probe, JC-1 (Molecular Probes). HepG2 cells were seeded to glass coverslips and cultured at least overnight before the experiment. After the indicated treatment, cells were washed twice in ice-cold PBS, and then incubated for 5 min at 37°C in PBS containing 2 μM JC-1. When excited at 490 nm, the dye will emit green fluorescence at low **∆**Ψm and red at high **∆**Ψm. Following incubation, the cells were washed once with PBS, then were imaged with a Zeiss Axiovert 25 fluorescent microscope equipped with a ProgRes C12 Plus CCD camera using a 63x objective and epifluorescent illumination. All experiments were repeated three times.

### JC-1 assay for flow cytometry

JC-1 assay kit for flow cytometry was used to detect mitochondrial depolarization in cultured HepG2 cells (Invitrogen, Molecular Probes, Hungary). After the indicated treatment, cells were incubated in the presence of 2 μM JC-1 for 5 min. Cells were washed once with PBS, then were immediately analyzed by a BD FacsCalibur flow cytometer (BD Biosciences, USA). Data were accumulated and reduced by Cellquest software (BD Biosciences, USA). JC-1 is accumulated in the mitochondria in a potential-dependent manner, indicated by a fluorescence emission shift from red (≈590 nm) to green (≈529 nm) upon depolarization. Consequently, mitochondrial depolarization is indicated by decrease in the red/green fluorescence intensity ratio. Cells in each category were expressed as percentage of the total number of stained cells counted. All experiments were repeated three times.

### Determination of apoptosis and necrosis with flow cytometry

Apoptosis was assessed after double staining with fluorescein isothiocyanate (FITC)-labeled annexin V (BD Biosciences, Hungary) and propidium iodide (PI; BD Biosciences, Hungary) using flow cytometry. First, the medium was discarded and the wells were washed twice with isotonic sodium chloride solution. Cells were removed from the plates using a mixture of 0.25% trypsin, 0.2% EDTA, 0.296% sodium citrate, 0.6% sodium chloride in distilled water. This medium was applied for 15 min at 37°C. Removed cells were washed twice in cold PBS and were resuspended in binding buffer containing 10 mM Hepes NaOH, pH 7.4, 140 mM NaCl, 2.5 mM CaCl_2_. Cell-count was determined in Burker's chamber. 100μL of buffer containing 10^5^ cells was transferred into 5 ml round-bottom polystyrene tubes. Cells were incubated for 15 min with FITC-conjugated annexin V and propidium iodide (PI). After this period of incubation, 400 μl of annexin-binding buffer (BD Biosciences, Hungary) was added to the tubes as described by the manufacturer. The samples were immediately measured by BD FacsCalibura a flow cytometer. Results were analyzed by Cellquest software (BD Biosciences, USA). Quadrant dot plot was introduced to identify living and necrotic cells and cells in early or late phase of apoptosis. Necrotic cells were identified as single PI-positive. Apoptotic cells were branded as annexin V-FITC-positive only and cells in late apoptosis were recognized as double-positive for annexin V-FITC and PI. Cells in each category were expressed as percentage of the total number of stained cells counted. All experiments were repeated three times.

### Cell cycle analysis with flow cytometry

Cell cycle analysis was performed using HepG2 cell line. HEpG2 cells were synchronized by serum deprivation prior to experiments in incomplete culture media overnight. Following treatments, the samples were fixed and permeabilized in 75% alcohol. The samples were washed and incubated in the staining media (PBS containing 10 μg/ml PI and 100 μg/ml RNAse A) for 1 h at 4°C. DNA content of the cells was measured on a FACSCalibur flow cytometer. Data were analyzed by FCS Express Version 3 software. All experiments were repeated three times.

### Statistical analysis

Values are presented as means ± SEM. Data were analyzed using 1-way ANOVA followed by Bonferroni’s test. Differences were considered significant at P < 0.05.

HepG2 cells were treated with 1000 times diluted DMSO as vehicle control (0) or with 0.1-10 μM 2,4-dimethoxyphenyl-*E*-4-arylidene-3-isochromanone (IK11) (**A**) for 24 hrs. Viability of the cells (**B**) was determined by MTT method in 96-well plates. Data are expressed as means ± SEM of three independent experiments running in six parallels. Small case Latin letters above the bars indicate significant differences. Means for a variable without a common letter differ, P < 0.05.

Alternatively, HepG2 cells were cultured until confluency in 6-well plates, then were treated or not (CTRL) with 1 μM IK11 for 24 hrs. Cell migration (**C**) was determined by comparing microscopic images of a monolayer wound taken at the beginning (0 h) and the end (24 h) of the incubation period. Representative images are shown; results of the 3 sets of independent experiments were basically identical.

Another aliquot of HepG2 cells were treated or not (open bars) with 0.5 μM (grey bars) or 1 μM (black bars) IK11 for 24 hrs in 6-well plates. Percentage of cells in G1, S and G2 phase of their cycle (**D**) was assessed by measuring their respective DNA content using a FACS Calibur flow cytometer. Results are expressed in percentages of the total number of cells means ± SEM of three independent experiments.

 Small case Latin letters above the bars indicate significant differences. Means for a variable without a common HepG2 cells were treated with 1000 times diluted letter differ, P < 0.05.

HepG2 cells were treated with 1000 times diluted DMSO as vehicle control (CTRL) or with 10 μM IK11 for 24 hrs. HepG2 cells were treated with DMSO as vehicle control (CTRL) or with 10 μM IK11 for 24 hrs. Apoptosis and necrosis was determined by flow cytometry after double staining the cells with FITC-Annexin V and propidium iodide. Representative dot-plots (**A**) are shown; results of the 3 sets of independent experiments were basically identical. Data combined from all experiments are expressed as percentages of the total number of cells, HepG2 cells were treated with 1000 times diluted and presented in pie chart (**B**).

HepG2 cells were treated with 1000 times diluted DMSO as vehicle control (CTRL) or with 10 μM IK11 for 30 min. JC-1 assay kit for flow cytometry (**A**) was used to detect mitochondrial depolarization in cultured HepG2 cells by measuring red (black) and green (grey) fluorescence intensity. Representative dot-plots are shown; results of the 3 sets of independent experiments were basically identical. Data combined from all experiments are expressed as fluorescent intensity in arbitrary units (a.u.), means ± SEM.

Mitochondrial depolarization was also demonstrated by using JC-1 fluorescent imaging (**B**). HepG2 cells were seeded to glass coverslips and cultured at least overnight before treating them or not (CTRL) with 10 μM IK11 for 24 hrs. Representative images are shown; results of the 3 sets of independent HepG2 cells were treated with 1000 times diluted experiments were basically identical.

HepG2 cells were treated with 1000 times diluted DMSO as vehicle control (0.0) or with 0.1-10 μM IK11 for 24 hrs. Dose–response of IK11 on intracellular ROS production (**A**) was determined by measuring fluorescent intensity of C-400 oxidized by the ROS.

Another aliquot of HepG2 cells were treated with 1000 times diluted DMSO as vehicle control (CTRL), 10 μM IK11 or 2 mM NAC (ROS scavenger) in different combinations as indicated for 24 hrs. Intracellular ROS production (**B**) and cell viability (**C**) was determined by measuring fluorescent intensity of C-400 oxidized by the ROS or by the MTT method, respectively. Data are expressed as means ± SEM of three independent experiments running in six parallels.

Small case Latin letters above the bars indicate significant differences. Means for a variable without a common letter differ, P < 0.05.

HepG2 cells were treated with 1000 times diluted DMSO as vehicle control (−), 0.1-10 μM IK11 or 10 μM PJ34 (PARP inhibitor, applied 1 h before the IK11 treatment) in different combinations as indicated for 24 hrs. Alternatively, PARP was silenced by siRNA technique before exposure to IK11. Dose–response of IK11 on cell viability (**A**) and intracellular ROS production (**B**) in the presence and absence of PJ34 or PARP silencing was determined by the MTT method or by measuring fluorescent intensity of C-400 oxidized by the ROS, respectively. Data are expressed as means ± SEM of three independent experiments running in six parallels.

Small case Latin letters above the bars indicate significant differences. Means for a variable without a common HepG2 cells were treated with 1000 times diluted letter differ, P < 0.05.

HepG2 cells were treated with 1000 times diluted DMSO as vehicle control (CTRL), 10 μM IK11 or 10 μM of the PARP inhibitor PJ34 (applied 1 h before the IK11 treatment) in different combinations as indicated for 6 h. Phosphorylation of JNK and Akt (**A**) was analyzed from whole cell extracts by immunoblotting utilizing phosphorylation-specific primary antibodies. We used GAPDH as a loading control. Representative immunoblots as well as band intensity bar diagrams (**B**) of 3 independent experiments are presented. Band intensities determined by Image J software and normalized to band intensities of the loading control were expressed as means ± SEM.

HepG2 cells were treated with 1000 times diluted DMSO as vehicle control (CTRL), 10 μM IK11, 10 μM of the JNK inhibitor SP-600125, 32.54 μM of the Akt pathway inhibitor LY294002, 5 μM of the specific Akt inhibitor Akt-inh. IV. or 50 μM of the ROS scavenger, JNK and Akt inhibitor resveratrol (Resv.) in different combinations as indicated for 24 hrs. Effect of IK11 on cell viability (**C**) in the presence and absence of the inhibitors was determined by the MTT assay. Data are expressed as means ± SEM of three independent experiments running in six parallels.

Another aliquot of HepG2 cells were treated (PJ34) or not (CTRL) with 10 μM of PJ34 for 24 hrs in 6-well plates. Percentage of cells in G1 (black bars), S (open bars) and G2 + M (grey bars) phase of their cycle (**D**) was assessed by measuring their respective DNA content using a FACS Calibur flow cytometer. Results are expressed in percentages of the total number of cells means ± SEM of three independent experiments.

Small case Latin and Greek as well as capitalized Latin letters above the bars indicate significant differences. Means for a variable without a common letter of the same case HepG2 cells were treated with 1000 times diluted and type differ, P < 0.05.

## Results

### IK11 inhibited migration, arrested cell cycle and induced death of HepG2 carcinoma cells

**Figure 1:**
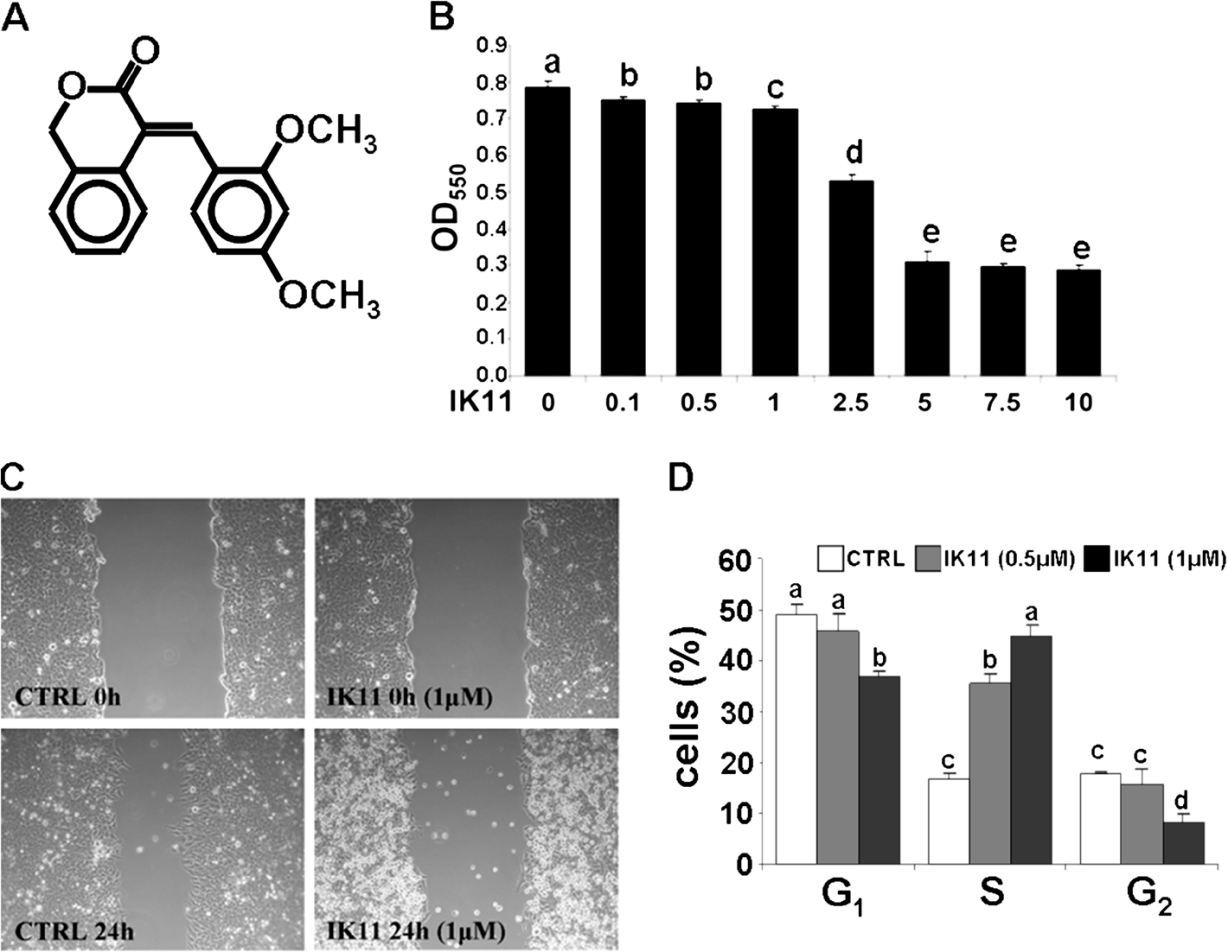
Effect of IK11 on cell migration, cell cycle and cell death.

It was previously demonstrated, that IK11 (Figure 1A) effectively killed A431 epidermoid carcinoma cell line [19]. To check its cytotoxicity on another tumor cell line, we determined its dose response on HepG2 human hepatocellular carcinoma cells. We found that it killed HepG2 cells in a concentration dependent manner in the range of 0.1 to 10 μM (Figure 1B) with the EC_50_ value of 3.50 ± 0.68 μM. Interestingly, higher concentrations of IK11 up to 25 μM did not increase cell death significantly (data not shown) by the end of the 24 h incubation time. We investigated the effect of IK11 on cell migration at a concentration (1 μM) in which it induced only a slight cell death (5%) by using a monolayer wound healing assay. When the cells were incubated in 1000 times diluted DMSO as vehicle control for 24 hrs, microscopic images showed a marked decrease in the width of the wound made by a pipette tip in confluent monolayer of cells indicating strong migration. In the presence of 1 μM IK11, the width of the wound remained almost identical to its starting value (Figure 1C) indicating that IK11 effectively inhibited migration of HepG2 cells at a concentration that caused only a slight cell death. Latter effect is demonstrated by the higher amount of floating debris in IK11 treated plates. We analyzed effect of 0.5 and 1 μM IK11 on the cell cycle. HepG2 cells were synchronized by overnight serum deprivation, treated with 0.5 or 1 μM IK11 for 24 hrs, then DNA content of the cells was determined by flow cytometry following propidium iodide staining. A concentration dependent decrease was observed in the number of G2 phase cells after the treatment with IK11 indicating that sublethal concentrations of IK11 prevented the entry of the cells into their G2 phase (Figure 1D).

### IK11 induced both apoptosis and necrosis in HepG2 carcinoma cells

**Figure 2:**
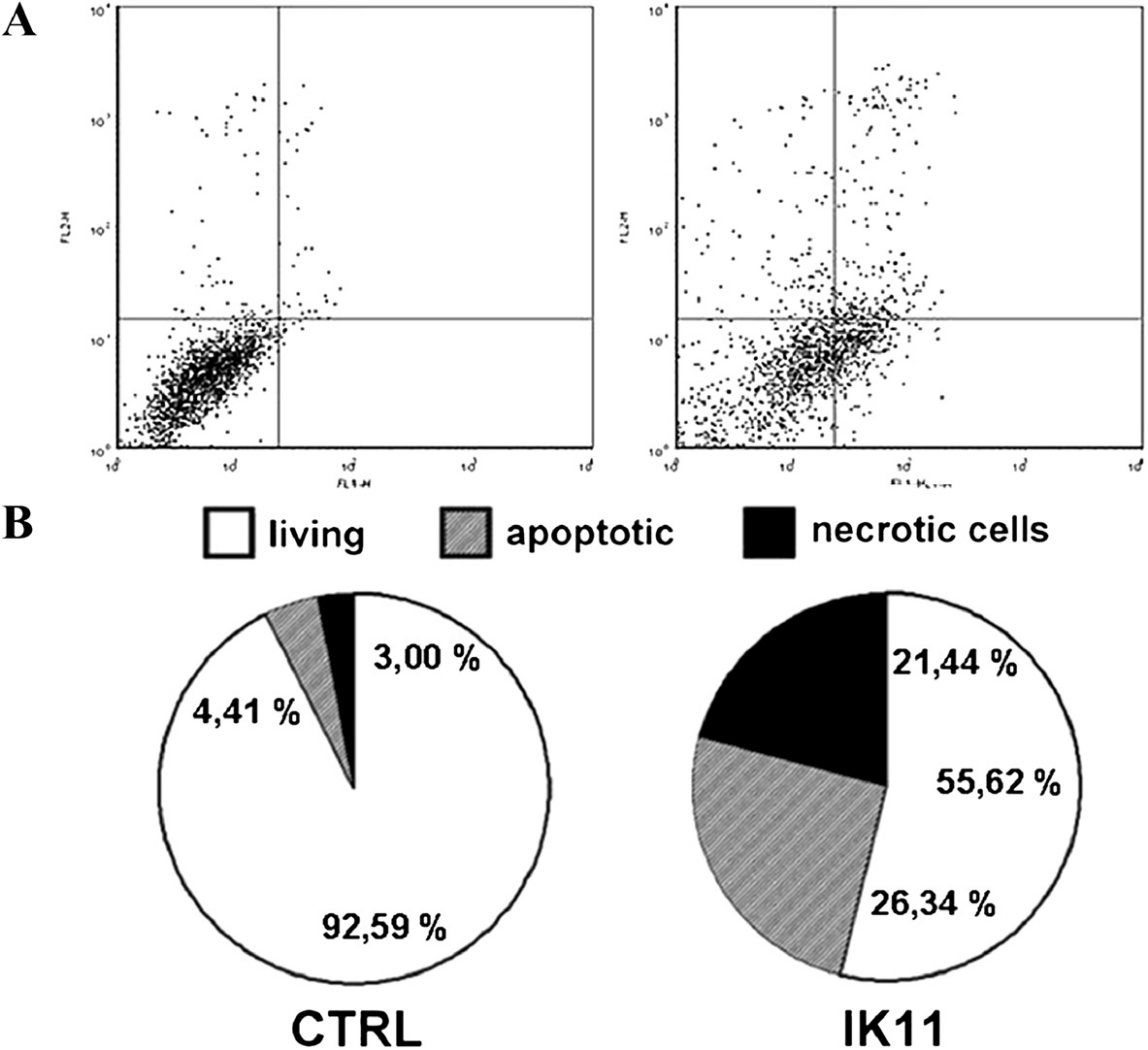
Effect of IK11 on apoptosis and necrosis.

We determined apoptosis and necrosis by flow cytometry following double staining of IK11-treated and untreated cells with FITC-conjugated Annexin V and PI. We found 26.34% of apoptotic and 21.44% of necrotic cells upon 24 h IK11 treatment compared to 4.41% and 3.00% found in control cells, respectively (Figure 2A,B). That means that 10 μM IK11 induced a 5.972-fold and 7.147-fold increase in apoptosis and necrosis, respectively.

### IK11 depolarized the mitochondrial membrane of HepG2 carcinoma cells

**Figure 3:**
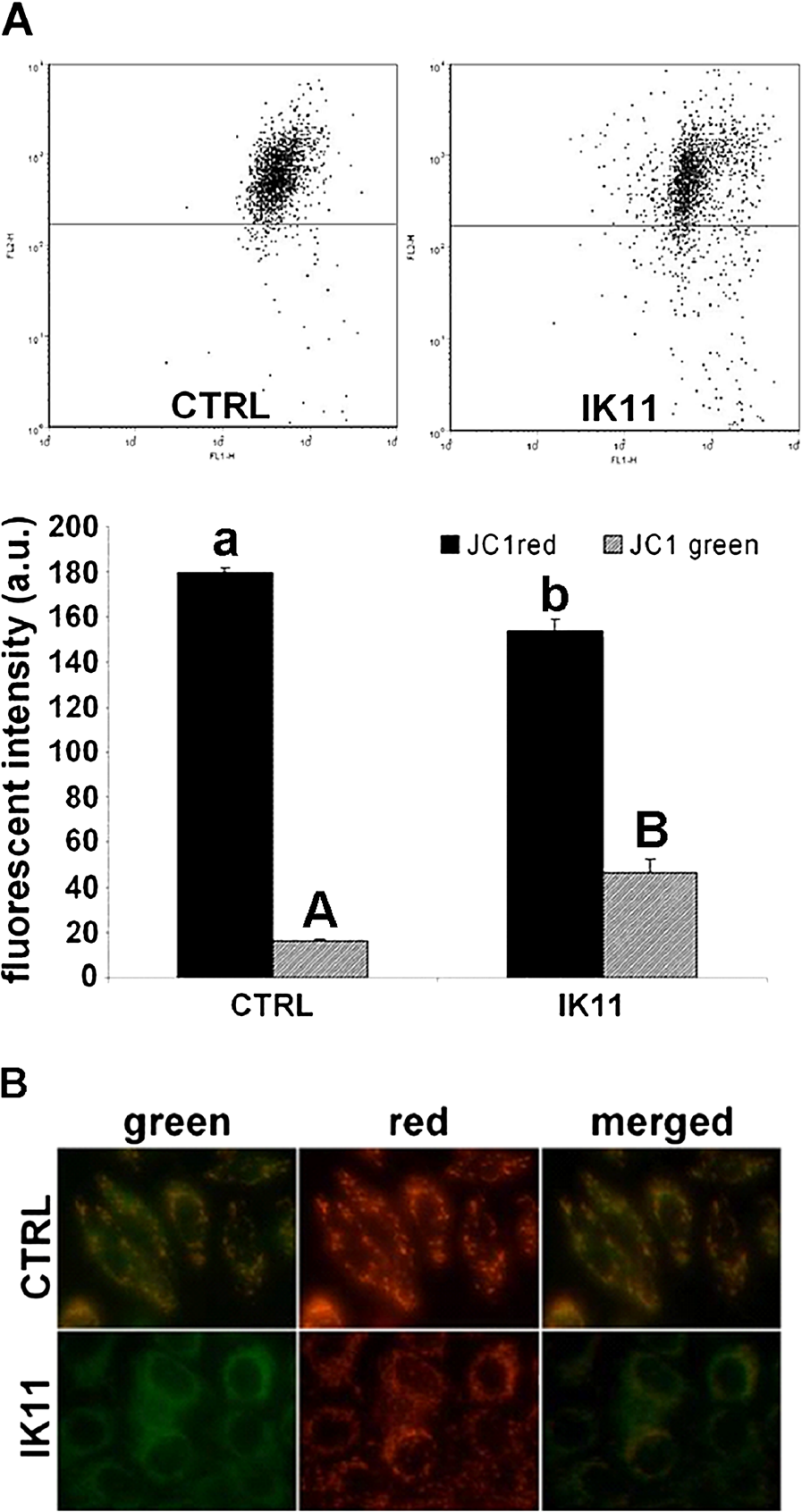
Effect of IK11 on the mitochondrial membrane potential.

Previously, it was found that IK11 induced caspase mediated apoptosis in A431 cells [19], and we detected both apoptosis and necrosis in IK11 treated HepG2 cells (Figure 2B,C). Since mitochondrial depolarization can be the consequence as well as the cause of both apoptotic and necrotic cell death [22], we studied mitochondrial membrane potential of control and IK11-treated (10 μM) cells 30 min after the start of treatment. After the treatment, cells were loaded with the voltage-sensitive fluorescent mitochondrial dye, JC1, then red and green fluorescence was determined by flow cytometry (Figure 3A) or fluorescent microscopic images of the same field were taken in the red and green channel (Figure 3B). Red to green shift of JC1 fluorescence upon IK11 treatment detected by both methods indicated that the drug induced depolarization of the mitochondria as soon as 30 min after its application.

### N-acetylcysteine (NAC) abolished IK11-induced ROS production, but hardly protected against death of HepG2 carcinoma cells

**Figure 4:**
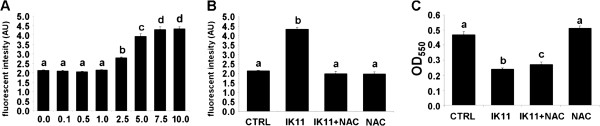
Effect of IK11 and NAC on endogenous ROS production and cell death.

Mitochondrial depolarization impairs the efficacy of the electron transport chain leading to massive ROS production. Therefore, we measured endogenous ROS production in IK11-treated and untreated HepG2 cells. We found, that IK11 induced ROS production in a concentration dependent manner in the range of 1 to 10 μM (Figure 4A) as it was determined by a C400 fluorescent assay following a 24 h incubation. To check whether ROS was a major mediator of IK11-induced cell death, we investigated the effect of 2 mM NAC on ROS production and cell death induced by the drug. We found that NAC abolished IK11-induced ROS production (Figure 4B), however, only slightly increased viability of IK11 treated HepG2 cells (Figure 4C). These observations suggested, that ROS production was a marginal mediator of IK11-induced cell death.

### PJ34 prevented cell death and decreased ROS production in IK11-treated HepG2 carcinoma cells

**Figure 5:**
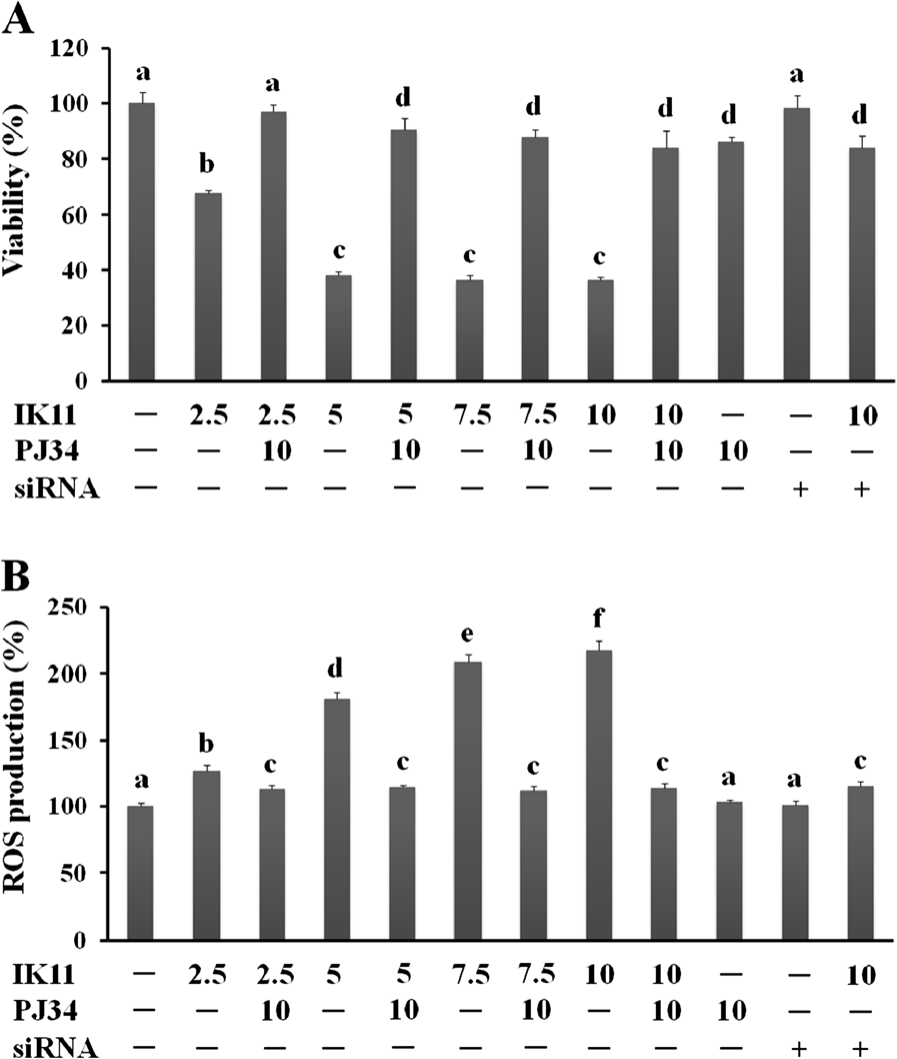
Effect of IK11 and PJ34 on endogenous ROS production and cell death.

To investigate whether PARP activation could participate in IK11-induced cell death, we applied three effective PARP-inhibitors of different chemical structure as well as silenced expression of the PARP gene by small interfering RNA (siRNA) technique before incubating the cells with IK11, and determined cell viabilities as well as endogenous ROS production after 24 h incubation. We found that 10 μM PJ34 almost completely prevented IK11-induced cell death (Figure 5A). Effect of HO3089 and L2286, the two other PARP inhibitors on IK11-induced cell death was similar to that of PJ34 (data not shown). Furthermore silencing of PARP also diminished IK11-induced cell death in about the same extent as the PARP inhibitors did (Figure 5A) indicating that it was most probably mediated via a PARP activation dependent mechanism. In addition to its effect on cell death, PJ34 significantly attenuated ROS production induced by IK11 (Figure 5B), although PJ34 does not have any ROS scavenging property. The other two PARP inhibitors (data not shown) and silencing of PARP (Figure 5B) diminished IK11 induced ROS production similarly to PJ34.

### Involvement of kinase signaling pathways in IK11-induced death of HepG2 carcinoma cells

**Figure 6:**
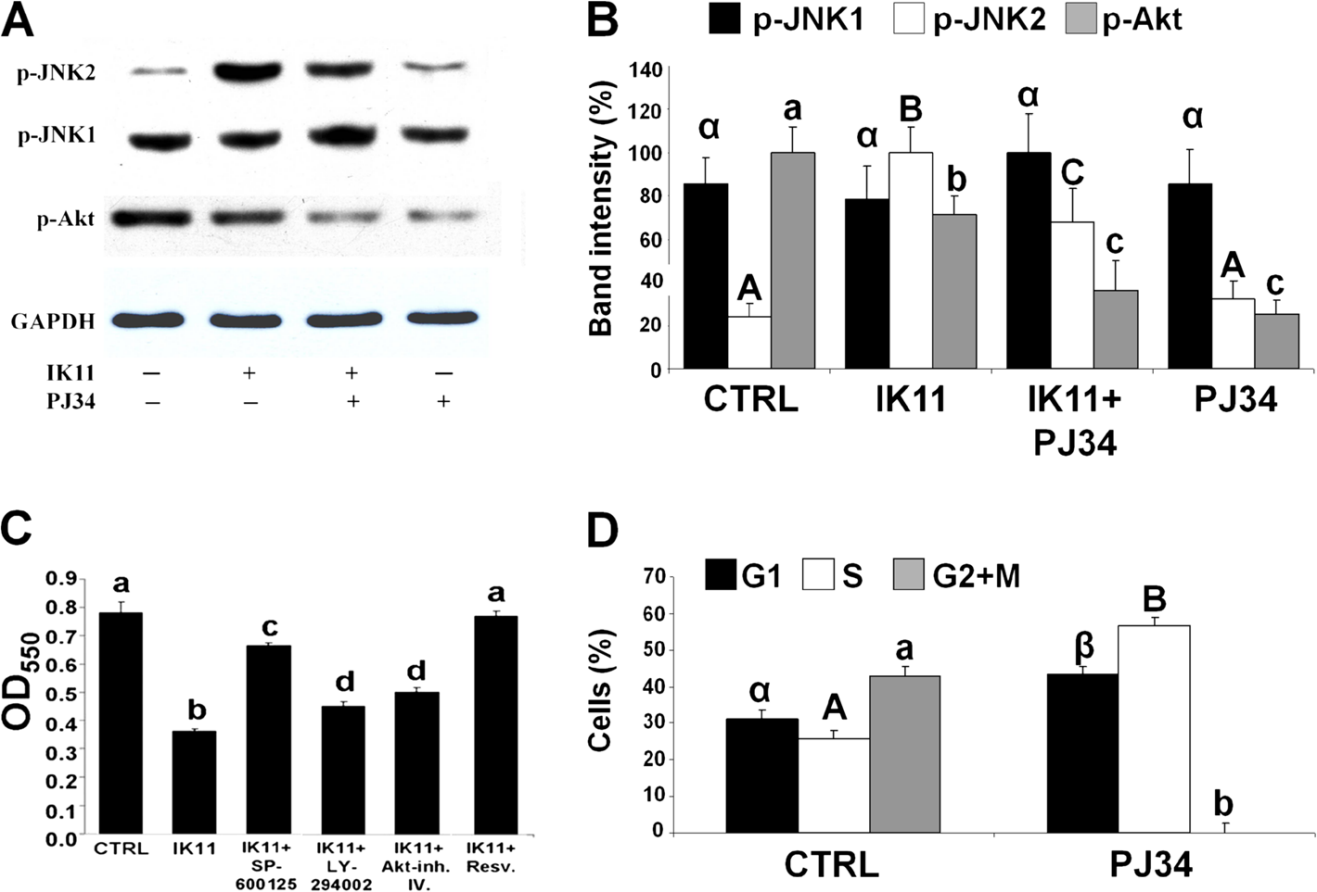
Effect of IK11 and different inhibitors on the kinase signaling pathways and cell death.

Many kinase signaling pathways including MAPKs and Akt were shown to be involved in PARP-mediated cell death [23]. Therefore, we investigated involvement of these kinase cascades in the mechanism of IK11-induced cytotoxicity. We observed activation of MAPKs but JNK1 as early as 10 min after application of 10 μM IK11 as it was revealed by immunoblotting utilizing phosphorylation-specific primary antibodies (data not shown). In case of Erk1/2 and p38, this increased phosphorylation did not increase any further (data not shown), while in case of JNK2, activation increased considerably during the following 6 h of incubation (Figure 6A,B). JNK1 activation was not affected by IK11 while activation of Akt was significantly reduced by IK11 (Figure 6A,B). For verifying the involvement of PARP activation in induction of JNK2 by IK11, we applied 10 μM PJ34 in combination with the 10 μM IK11 for 6 h, then assessed kinase activation by immunoblotting. We found that PJ34 inhibited IK11-induced activation of JNK2 and further reduced Akt phosphorylation (Figure 6A,B). PJ34 in the absence of IK11 did not affect JNK1 and JNK2 activation significantly, while it reduced Akt phosphorylation more strongly than IK11 did (Figure 6A,B). In order to establish physiological significance of involvement of these kinases in IK11-induced cell death, we applied inhibitors of them in combination with IK11, and determined viability by using MTT assay of the HepG2 cells 24 h after. We found that p38 and ERK inhibitors did not affect (data not shown), while inhibition of Akt by two different inhibitors only moderately attenuated the cytotoxicity of IK11 (Figure 6C) indicating that activation of the Akt pathway could at least partially mediate IK11-induced death of HepG2 carcinoma cells. Inhibition of JNK pathway diminished the effect of IK11 in about the same significant extent as PJ34 did (compare Figures 5A and 6C) suggesting that JNK2 activation could be a major mediator in the cytotoxicity of IK11. Cytotoxicity of IK11 was abolished by trans-resveratrol that has various, mostly cytoprotective effects - in the concentration used by us - including ROS scavenging as well as inhibition of JNK and Akt (Figure 6C). In combination with the data in Figure 4C, these data suggest that JNK2 activation was most likely responsible for the IK11-induced death of HepG2 carcinoma cells, however, combined inhibition of JNK, Akt and ROS was necessary to completely protect against it.

Contrary to previous studies utilizing oxidative stress models, we found that phosphorylation of Akt in untreated HepG2 cells was high, and PJ34 drastically decreased it (Figure 6A,B). Therefore, we investigated the effect of PJ34 treatment on the cell cycle of HepG2 cells. As it is presented on Figure 6D, PJ34 prevented entry of the cells into G2 phase of their cycle similarly as IK11 did (Figure 1D).

## Discussion

IK11 was previously found to kill A431 epidermoid carcinoma cells effectively [19]. In the present report we determined its cytotoxic effect in the HepG2 human hepatocellular carcinoma line. We found that IK11 at the concentration of 1 μM prevented proliferation and entry of the cells into their G2 phase. At higher concentrations it killed the cells in a concentration dependent manner by increasing both apoptosis and necrosis.

Mitochondria can promote cell death by various mechanisms depending on the severity of the stimulus to the mitochondrial membrane systems [15]. Depolarization that leads to permeabilization of the outer mitochondrial membrane could result in release of intermembrane pro-apoptotic proteins including cytochrome c and apoptosis inducing factor, causing caspase dependent and independent apoptosis, respectively. On the other hand, permeabilization of both membranes, e.g., by opening the permeability transition pore, can lead to ATP depletion, swelling and disruption of mitochondria, and finally to necrotic cell death [15]. By using two different methods, we demonstrated that IK11 induced depolarization of the mitochondrial membrane as early as 30 min after its application, suggesting that both apoptotic and necrotic cell death induced by the drug could be mitochondria-mediated.

Impaired mitochondrial integrity causes malfunctioning of the respiratory chain that in turn produces substantial amount of ROS mainly at the level of cytochrome oxidase [15]. Accordingly, we determined the effect of IK11 on ROS production, and found that it induced production of ROS in a concentration dependent manner that correlated with the dose response of the drug on cell death. This suggested that the killing of the hepatocellular carcinoma cells could have been mediated by ROS originating from malfunctioning mitochondria. However, in that case, elimination of ROS by high concentration of NAC should have protected the HepG2 cells from IK11-induced death. As we found, NAC indeed eliminated IK11-induced ROS production, but only slightly diminished cell death, indicating ROS was only a marginal mediator. In contrast, PJ34, an effective inhibitor of PARP that does not have any ROS-scavenging property, diminished both ROS production and cell death induced by 2.5-10 μM IK11 almost to the level of untreated cells. Furthermore, silencing of the expression of PARP gene by siRNA attenuated IK11 induced ROS production in about the same extent as PJ34 and two other PARP inhibitors did. These results clearly verified that ROS-production was a phenomenon accompanying cell death rather than a major mediator of it under our experimental conditions. Furthermore, it indicated significant involvement of PARP-activation in IK11-induced death of the hepatocellular carcinoma cells.

Activation of MAPK and the PI3K-Akt pathways is considered to be directly or indirectly involved in extra-nuclear effects of PARP-activation [8,24,25]. MAPK pathways, which consist of the ERK1/2 the JNK and the p38 pathways, govern cell proliferation, differentiation, stress responses, and survival, among other functions. JNK and p38 were found to be involved in oxidative stress, inflammatory conditions, cytokine production, and can induce apoptosis under numerous experimental conditions. In contrast, Erk1/2 is thought to possess both apoptotic and antiapoptotic properties and it was shown to be involved in the regulation of cell migration [26]. Additionally, it has been suggested that JNK has targets in the mitochondria and that mitochondrial JNK activation in response to ROS causes cytochrome c release and cell death [27]. Akt/PKB plays a key role in multiple cellular processes such as cell proliferation, apoptosis and cell migration, oxidative stress and it is thought to be involved in survival pathways by inhibiting apoptotic processes, e.g., via inhibition of the MAPK pathways. Similarly to ERK, the PI3K-Akt pathway is considered to be cytoprotective, although in certain systems it was found to mediate apoptosis [28]. Accordingly, many compounds possessing apoptotic properties were found to activate the MAPK pathways and inhibit the PI3K-Akt pathway, leading to decreased cell migration and proliferation or to the death of the cell.

Under our experimental conditions, we found activation of the MAPKs but JNK1 as early as 10 min after the addition of IK11. Since their activation preceded mitochondrial depolarization, early activation of these signaling kinases could trigger the mitochondrial-dependent death of hepatocellular carcinoma cells. Out of the kinases studied, JNK2 activation was sustained during longer incubation with IK11. Interestingly, JNK2 but not JNK1 activation was found to be involved in high arsenite concentration induced DNA damage mediated apoptosis [29]. The similar pattern of JNK activation suggests that IK11 induced death of HepG2 cells could be mediated by DNA damage that could trigger PARP activation leading to mitochondrial depolarization mediated cell death. Attenuation of JNK2 activation by PJ34 as well as failure by NAC to protect against IK11 induced cell death, support such mechanism.

Very recently, a strong correlation was found between Akt activation and increased proliferation potential of HepG2 cells [30]. Accordingly, we found quite high activation of Akt in untreated HepG2 cells that was diminished by IK11 and PJ34 too. These results are consistent with recent reports [28,29], however, contrary to those that we and others found in oxidative stress models indicating again that the mechanism of IK11 induced cell death could differ considerably from that of oxidative stress.

To verify the exact role of the kinase pathways, we applied selective inhibitors of them and measured IK11-induced cell death in their presence. We found that pharmacological inhibition of PI3K-Akt pathway only moderately increased survival of IK11-treated cells while that of p38 and ERK did not have any significant effect. On the other hand, the JNK-inhibitor SP-600125 significantly, and trans-resveratrol completely, protected the hepatocellular carcinoma cells from the toxic effects of IK11. These results are significant in several aspects.

First, PI3K-Akt is considered antiapoptotic kinase signaling pathway since it was previously found to be active in several human cancers and its inappropriate activation or inhibition resulted in blocked proliferation and enhanced sensitivity toward cytotoxic agents. One major mechanism by which this antiapoptotic effect is mediated is phosphorylation of Ser^83^ of apoptosis signal-regulating kinase 1 (ASK1) by Akt, rendering this pro-apoptotic kinase inactive [31]. However, Akt-mediated phosphorylation of ASK1 at Thr^838^ activates ASK1 and causes the subsequent downstream activation of p38 MAPK, leading to apoptosis [31]. We found inhibition of Akt upon IK11 treatment and moderately increased cell survival when the PI3K-Akt pathway was blocked at two different levels, namely at PI3K and Akt by two separate selective inhibitors, indicating that this pathway was a minor mediator of IK11-induced cell death. Furthermore, we found that PJ34 inhibited the entry of HepG2 cells into the G2 phase of their cycle similarly as IK11 did that could be the result of attenuated activation of Akt by these substances.

Second, activation of JNK is thought to trigger mitochondrial depolarization-mediated apoptotic cell death [27]. In a complete agreement with this result, we found that inhibition of JNK2 substantially inhibited the death of hepatocellular carcinoma cells induced by IK11.

Third, we found that inhibition of PARP decreased activation of JNK2 and prevented IK11-induced cell death, suggesting a strong relationship between activation of PARP and JNK2. In a recent separate study, we found that early activation of MAPKs, mediating cell death in oxidative stress, is compensated by increased expression of MKP-1, and inhibition of PARP augmented MKP-1 expression, resulting in cytoprotection, which was prevented by silencing MKP-1 [13]. It is likely that a similar mechanism was involved in the observed correlation between PARP and JNK2 activation in the IK11-induced, mitochondrial depolarization-mediated death of hepatocellular carcinoma cells.

Finally, the observation that trans-resveratrol abolished the cytotoxic effect of IK11 indicated that Akt and JNK2 activation as well as ROS production were all involved in this effect, although JNK2 activation seemed to be the major mediator of it.

## Conclusion

Activation of JNK2 led to mitochondrial depolarization-mediated necrotic and apoptotic death of IK11-treated hepatocellular carcinoma cells that were prevented to different extents by inhibitors of Akt, JNK and PARP. These results indicate a pro-apoptotic role of Akt in this system, and raise attention to a novel mechanism that should be considered when cancer therapy is augmented with PARP-inhibition, namely cytoprotection by inhibition of JNK2.

## Abbreviations

IK11, 2,4-Dimethoxyphenyl-E-4-arylidene-3-isochromanone; JNK, C-Jun N-terminal kinase; ERK1/2, Extracellular signal regulated kinase 1/2; PKB/Akt, Protein kinase B; MAPK, Mitogen activated protein kinase; ROS, Reactive oxygen species; PI3K, Phosphatidylinositol 3 kinase; MKP-1, MAPK phosphatase-1; NAC, N-acetylcysteine; PJ34, N-(6-Oxo-5,6-dihydro-phenanthridin-2-yl)-N,N-dimethylacetamide.

## Competing interests

The authors declare that they have no competing interests.

## Authors’ contributions

LT synthesized the compound IK11 and made original observations leading to this work. GF Jr and SB designed the work and GF Jr contributed to the critical revision of the script. EP conducted cell cycle analyses, RB measured apoptosis and necrosis and mitochondrial depolarization with flow cytometry. VB and TZ accomplished fluorescent microscopy, AC, PJK performed immunoblot analysis and TZ made the wound healing assay. RB contributed to the conception and design of the entire study, performed the rest of the experiments and wrote the initial drafts of the manuscript. All authors read and approved the final manuscript.
